# Are saving appearance responses typical communication patterns in Alzheimer's disease?

**DOI:** 10.1371/journal.pone.0197468

**Published:** 2018-05-23

**Authors:** Masateru Matsushita, Yusuke Yatabe, Asuka Koyama, Akiko Katsuya, Daisuke Ijichi, Yusuke Miyagawa, Hiroto Ikezaki, Noboru Furukawa, Manabu Ikeda, Mamoru Hashimoto

**Affiliations:** 1 Center for Medical Education and Research, Faculty of Life Sciences, Kumamoto University, Kumamoto, Japan; 2 Mental Health and Welfare Center, Department of Health and Welfare, Kumamoto Prefectural Government, Kumamoto, Japan; 3 Department of Neuropsychiatry, Faculty of Life Sciences, Kumamoto University, Kumamoto, Japan; 4 Department of Rehabilitation, Atsuchi Neurosurgical Hospital, Kagoshima, Japan; 5 Department of Speech-Language-Hearing Science, Kumamoto Health Science University, Kumamoto, Japan; 6 Department of Psychiatry, Osaka University Graduate School of Medicine, Suita, Japan; Nathan S Kline Institute, UNITED STATES

## Abstract

**Introduction:**

To keep up appearances, people with dementia sometimes pretend to know the correct answer, as seen during administration of neuropsychological tests such as the Mini-Mental State Examination (MMSE). These saving appearance responses (SARs) of people with dementia often lead to caregivers and/or medical staff underestimating the severity of dementia and impede proper early initiation of treatment. However, most descriptions of SARs are based on empirical knowledge of clinicians. In this study, we investigated whether SARs are typical communication patterns in people with Alzheimer’s disease (AD), compared with mild cognitive impairment (MCI) or dementia with Lewy bodies (DLB).

**Methods:**

The participants were 107 outpatients with AD, 16 with mixed AD with cerebrovascular dementia, 55 with MCI, and 30 with DLB. We assessed the occurrence of SARs during the MMSE. The relationships between the SARs and AD were examined by the *χ*^2^ test and logistic regression analysis.

**Results:**

People with AD who showed SARs were 57.9%, whereas those with MCI were 18.2% and DLB were 20.0% (*P*_with Bonferroni correction_ < 0.05). Although there were significant differences in some variables in each group of diagnosis, logistic regression analysis showed that people with AD were more likely to show SARs than those with MCI (Odds ratio = 3.48, 95% Confidential Interval = 1.18–10.28) and DLB (Odds ratio = 4.24, 95% Confidential Interval = 1.50–12.01), even after controlling for sex, estimated disease duration, MMSE, and frontal assessment battery scores.

**Conclusion:**

The occurrence of SARs could be found most frequently in people with AD. Clinicians should develop a respectful attitude toward dementia patients with SARs because SARs imply conflicted feelings about questions that patients cannot answer correctly.

## Introduction

In general, people usually wish to suppress negative information in order to not suffer a disadvantage or face public shame. These saving appearance responses/behaviors (SARs) are an attempt to maintain a normal external and superficial appearance to gloss over a mistake or evade responsibility. As another example, people with socialized personalities sometimes avoid a direct answer in order to communicate with others smoothly. In Japan, we refer to this type of behavior of keeping up appearances behavior as ‘*toritsukuroi*’ or ‘*toritsukurou*.’

SARs can also be seen in clinical practice when physicians see people with suspected dementia [1]. For example, if a physician asks a patient about recent news, some patients will say “I seldom watch TV” and dodge the question. In other instances, if a clinical psychologist asks a patient the name of the patient’s primary doctor, some patients respond, “I usually call him doctor” and do not answer either “I know” or “I don’t know.” Because these SARs sometimes cause caregivers and physicians to underestimate the severity of dementia, early detection and treatment of dementia is impeded.

SARs can be considered as resulting from a patient’s dilemma about memory loss. Although dementia is commonly accompanied by memory impairment, SARs are likely to cover for the impaired function by preserving the ability to communicate [2]. Caregivers and physicians must know about and watch for these kinds of reactions and should pay attention with a respectful and gentle attitude.

In one of the few studies in this area, Fujisawa et al. reported that the SARs could be seen in mild cognitive impairment (MCI), an early stage of Alzheimer’s disease (AD), and gradually faded away as the severity of dementia became more severe [1]. Thus, SARs might be an early sign of Alzheimer’s disease. In a previous study, we studied SARs during the neuropsychological examination “Mini-Mental State Examination” (MMSE) [3]. Because Matsuda wrote that SARs differ depending on the situation and the person interacting with the patients with dementia [2], we studied SARs in a semi-structured examination setting and reported that SARs can be classified by following 6 categories: 1) “I can’t answer a sudden question” 2) Responses that imply a dislike or weakness about something 3) “I don’t do/I’ve never done such a thing” 4) “I remembered until just a while ago” 5) “I don’t care/I am not aware of it” 6) Other. Based on our qualitative research [3], we developed an observational assessment-tool for the SARs (See S1 Appendix). This observational assessment, which can be performed in conjunction with the MMSE, is expected to be widely available for medical professionals in the field of psycho-geriatrics and gerontology.

Until now, SARs have been discussed in association with AD. Tanabe et al. indicated that in the overall behavioral changes of dementia, Pick’s disease is characterized by a ‘going my own way’ type of behavioral pattern, whereas AD is characterized by keeping up appearances of faking one’s way through a situation [4]. Although a previous study by Fujisawa et al. compared the occurrence of SARs among the people with AD, MCI, and healthy elderly people [1], no study has been reported to compare SARs between people with AD and other types of dementia. If SARs are a clinical sign of AD, it is essential to examine whether SARs are more common in people with AD than in other type of dementia. People with dementia with Lewy bodies (DLB) which is the second most common cause of neurodegenerative dementia after AD in people aged over 65 years possess properties differing from AD. Although comparing the SARs in AD with DLB allow us to discuss the pathophysiological aspect of AD in more detail, to our knowledge, no studies have compared SARs between people with AD and those with DLB. Furthermore, there is only one study compared SARs of AD with MCI which is a potential precursor to AD [1]. Therefore, the purpose of this study was to examine whether SARs are a typical symptom of people with AD by comparing SARs in AD, DLB, and MCI.

## Methods

### Procedures

The ethics and research committees of Kumamoto University approved this study. We obtained written informed consent from all patients and their primary caregivers before starting the study. The participants were 208 outpatients who visited the Memory Clinic at Kumamoto University Hospital from October 2016 to September 2017. The mean age of the patients was 77.9 ± 8.61 years, and 70.7% of the patients were female. The mean scores of the MMSE and Frontal Assessment Battery (FAB) were 19.9 ± 6.13 and 11.01 ± 3.71, respectively. This study had 107 people with AD, 55 with MCI, 30 with DLB, and 16 with AD mixed cerebrovascular disease. All patients were diagnosed by senior neuropsychiatrists. To diagnose the patients, we conducted neuropsychological tests including the MMSE and FAB, brain magnetic resonance imaging, I-123 iodoamphetamine (IMP) single-photon emission computed tomography, and laboratory tests. The patients with AD were diagnosed according to the criteria established by the National Institute of Neurological and Communicative Disorders and Stroke and the Alzheimer’s disease and Related Disorders Association [5]. To make other diagnoses, we used the national Institute of Neurological Disorders and Stroke and Association Internationale pour la Recherche te I’Enseignement en Neurosciences criteria for vascular dementia [6]; the published criteria on Mild Cognitive Impairment by Petersen [7]; and the Consortium on DLB International Workshop [8]. In this study, we performed cardiac 123I-MIBG scintigraphy, and detected decreased MIBG uptake in people with the presence of dementia and at least two of three core features, to define the people with DLB.

Highly-experienced medical professionals including psychiatrists, clinical psychologists, occupational therapists, and speech therapists conducted the MMSE [9,10] and FAB [11,12,13]. When the examiners asked patients with suspected dementia to answer the MMSE questions, they checked whether those with dementia exhibited SARs using the Japanese version of Toritsukuroi Assessment Battery (Ja-TAB) (S1 Appendix.). To avoid potential reporting bias, the primary investigator (M.M.) blinded observers to the name of the disease that caused dementia.

### Measurement

SARs were assessed using the Ja-TAB that allows the examiner to check the presence of SARs easily when the medical staff conducted the MMSE (S1 Appendix.). If the patient with suspected dementia tries to keep up appearances verbally, the examiner selects the category of the patient’s verbal response and checks off the boxes of response category and the number of SARs. The SARs do not include erroneous answers or responses meaning ‘I don’t know’ because the SARs are an attitude to cover one’s shortcomings without feeling the shame of a plausible misrepresentation.

The MMSE is one of the most frequently used screening tools to estimate general cognitive function and has been shown to have adequate reliability and validity [9,10]. The Japanese version of MMSE with a maximum score of 30-points consists of 10 domains of cognitive functions. A higher MMSE score reflects a more favorable cognitive function.

The FAB is a brief and reliable method to assess frontal executive functioning [11,12,13]. It is composed of the following 6 neuropsychological sub-tests: (1) similarities (conceptualization), (2) lexical fluency (mental flexibility), (3) motor series “Luria” test (programming), (4) conflicting instructions (sensitivity to interference), (5) Go-No Go (inhibitory control), (6) prehension behavior (environmental autonomy). Total score is from a maximum of 18, higher scores indicating better performance. The reliability and validity of the Japanese version of the FAB were well established by Kugo et al [12].

### Statistical analyses

In this study, we defined individuals who have one or more SARs as the “With SARs” group. Individuals who don’t have SARs were identified as the “Without SARs” group. The *χ*^2^ test, unpaired *t* test, and one-way analysis of variance (ANOVA) were used to examine between-group differences. For multiple comparisons, the Bonferroni correction and post-hoc Sidak test were applied. To compare the number of SARs between 4 groups, we applied the Kruskal-Wallis test with pairwise comparison. Logistic regression analysis was used to assess the relationship between the SARs and disease after controlling for other potential confounding factors. With regard to mixed dementia, we excluded this group from logistic regression analysis because the number of people was small. All statistical analyses were performed using IBM SPSS Statistics Version 20.0.0.1 statistical software (Tokyo, IBM Corporation, 2011). A 2-tailed *P* value of less than 0.5 was considered as significant.

## Results

**Fig 1:**
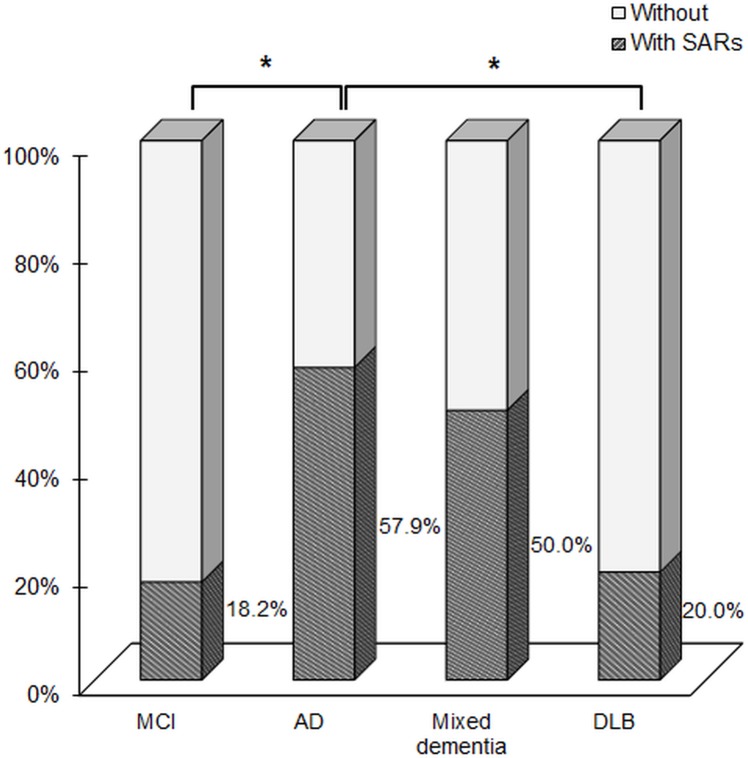
The percentages of people who had the saving appearance responses (SARs). MCI: mild cognitive impairment; AD: Alzheimer’s disease; DLB: dementia with Lewy bodies; *: *P* < 0.05 with Bonfferoni correction.

We found SARs in 86 of 208 patients (41.3%). 56.1% of the people with AD were observed to have given SARs (Fig 1). As shown in Fig 1, more people with AD (57.9%), compared to those with MCI (18.2%) or DLB (20.0%), showed SARs (*χ*^2^ = 30.5, *d*.*f*. = 3, *P* < 0.001). When we compared the total number of SARs, a Kruskal-Wallis test showed that there was a significant difference of median (*H* = 30.5, *d*.*f*. = 3, *P* < 0.001) and the null hypothesis was rejected. In pairwise comparisons, we found that the number of SARs in people with AD was significantly different to those with MCI (adjusted *P* < 0.001) and DLB (adjusted *P* = 0.001).

Comparing each type of SARs between 4 disease groups, there were significant differences of median in the types of “I can’t answer a sudden question” (*H* = 13.4, *d*.*f*. = 3, *P* = 0.004) and “I don’t care/I am not aware of it” (*H* = 22.2, *d*.*f*. = 3, *P* < 0.001). Regarding these 2 types of responses, people with AD had a significantly higher median of total number of SARs than those with MCI (adjusted *P* = 0.001) or DLB (adjusted *P* = 0.041).

Comparisons of demographics and neuro-psychological variables were summarized in Table 1. We found that there were significant differences in age (*P* = 0.004), sex (*P* = 0.004), estimated disease duration (*P* < 0.001), prescriptions of anti-dementia drugs (*P* <0.001) and anti-psychotic medication (*P* = 0.048), MMSE (*P* < 0.001), FAB (*P* < 0.001), and GDS (*P* = 0.042). Next, we examined whether the SARs were related to these variables and found significant differences in sex (*χ*^2^ = 8.13, *d*.*f*. = 1, *P* = 0.005), estimated disease duration (*t* = 2.64, *d*.*f*. = 206, *P* = 0.009), MMSE (*t* = -4.71, *d*.*f*. = 206, *P* < 0.001), FAB (*t* = -2.65, *d*.*f*. = 206, *P* = 0.009) and GDS (*t* = -3.81, *d*.*f*. = 206, *P* < 0.001) between the participants with and without SARs.

**Table 1:** Demographics of participants.

	MCI		AD		Mixed dementia		DLB		*F / Χ*^*2*^	*P*	Post-hoc Sidak test / *Z* test with Bonferroni correction
	Mean	SD	Mean	SD	Mean	SD	Mean	SD			
**Age** (years)	75.8	7.79	77.3	9.27	83.3	6.71	80.6	6.72	4.55	0.004**	MCI < Mixed dementia
**Male/female** (% of female)	23/32	(58.2%)	20/87	(81.3%)	5/11	(68.8%)	13/17	(56.7%)	12.85	0.004**	MCI, DLB < AD
**Education** (years)	12.0	2.24	11.6	2.14	10.6	2.50	11.6	2.49	1.69	0.17	*n*.*s*
**Estimated disease duration** (years)	3.5	2.09	5.2	2.24	4.4	2.68	4.4	2.96	6.65	< 0.001***	MCI < AD
**With anti-dementia drugs**	22	(41.8%)	98	(91.6%)	12	(75.0%)	21	(70.0%)	47.11	< 0.001***	MCI, DLB < AD
**With anti-psychotic medications**	6	(10.9%)	11	(10.3%)	1	(6.3%)	9	(30.0%)	9.23	0.048*	AD < DLB
**MMSE**	25.6	2.69	17.1	4.53	19.1	5.58	19.9	8.63	34.01	< 0.001***	AD, Mixed dementia, DLB < MCI
**FAB**	13.1	3.31	10.5	3.20	9.8	2.59	9.8	4.99	9.21	< 0.001***	AD, Mixed dementia, DLB < MCI
**GDS**	3.7	2.90	2.8	2.33	3.6	2.48	4.1	2.79	2.78	0.042*	*n*.*s*

MCI: mild cognitive impairment; AD: Alzheimer’s disease; DLB: dementia with Lewy bodies. MMSE: Mini-Mental State Examination; FAB: Frontal Assessment Battery; GDS: Geriatric Depression Scale.

***: P < 0.001

**: P < 0.01

*: P < 0.05

n.s: not significant.

Logistic regression analysis revealed that people with AD are more likely to show the SARs than those with MCI, as shown in Table 2 (Odds ratio = 3.48, 95% Confidential Interval = 1.18–10.28, Wald = 5.07, *d*.*f*. = 1, *P* = 0.024). In addition, we found that people with AD were more likely to have SARs than those with DLB (Odds ratio = 4.24, 95% Confidential Interval = 1.50–12.01, Wald = 7.39, *d*.*f*. = 1, *P* = 0.007), even after controlling for sex, estimated disease duration, MMSE, and FAB scores (Table 3).

**Table 2:** Results of logistic regression analysis for the relationship between saving appearance responses and AD in people with AD and MCI.

	Odds ratio	(95% CI)	Wald	*P*
**Sex**				
Male	*Reference*			
Female	1.77	0.77–4.07	1.82	0.178
**Estimated disease duration**	1.04	0.89–1.23	0.25	0.619
**MMSE**	0.94	0.84–1.04	1.52	0.218
**FAB**	1.01	0.88–1.15	0.01	0.920
**Diagnose**				
MCI	*Reference*			
AD	3.48	1.18–10.28	5.07	0.024*

MMSE: Mini-Mental State Examination; FAB: Frontal Assessment Battery; MCI: mild cognitive impairment; AD: Alzheimer's disease

*: *P* < 0.05.

**Table 3:** Results of logistic regression analysis for the relationship between saving appearance responses and AD in people with AD and DLB.

	Odds ratio	(95% CI)	Wald	*P*
**Sex**				
Male	*Reference*			
Female	1.70	0.69–4.18	1.34	0.247
**Estimated disease duration**	0.99	0.84–1.17	0.01	0.906
**MMSE**	0.92	0.83–1.01	2.88	0.090
**FAB**	1.05	0.91–1.22	0.42	0.517
**Diagnose**				
DLB	*Reference*			
AD	4.24	1.50–12.01	7.39	0.007**

MMSE: Mini-Mental State Examination; FAB: Frontal Assessment Battery; DLB: dementia with Lewy bodies; AD: Alzheimer's disease

**: *P* < 0.01.

## Discussion

The aim of this study was to compare SARs during MMSE among the people with AD, MCI, and DLB. Our results showed that 57.9% of people with AD displayed SARs, whereas 18.2% of those with MCI and 20.0% of those with DLB displayed SARs. As would be expected, our results made it clear that people with AD are more likely to show SARs compared with those with DLB and MCI, even after controlling for possible confounding factors. The high probability of people showing 2 types of SARs (“I can’t answer a sudden question”, “I don’t care/I am not aware of it”) in AD suggests that these types of SARs are a distinctive communication pattern in AD. SARs might be a suggestive sign of AD, although people with SARs are not always diagnosed as having AD, because the prevalence of SARs was 18.2% and 20.0% in individuals with MCI and DLB, respectively.

A previous report showed that SARs emerge at a high rate in people with MCI or AD compared to normal elderly [1]. SARs in AD then gradually decrease as the disease progresses [1]. We think that these facts are associated with the function of the frontal lobe. AD manifests itself as the progressive decline of orientation and memory, yet other cognitive functions such as empathy and insight are relatively preserved in the early stages of AD. SARs might be due to compensative behaviors of memory impairment using social communication skills. On the other hand, our results are different from a previous report on SARs of AD and MCI. Although it was found that there was a significant difference of SARs between MCI and AD in this study, Fujisawa et al. (2013) reported that the frequency of SARs in people with MCI did not differ from those with AD [1]. The differences of the results may be derived from that we adopted a different definition of SARs than this previous report. In the previous report, the definition of SARs was “the answering that someone does not remember recent news for the reason resulting from physical feature, when physician ask patient to tell recent news” (such as “I didn’t read newspaper due to poor eyesight”) [1]. This type of response is similar to the responses that imply a dislike or weakness about something in our definition. As noted earlier, two types of SARs such as “I can’t answer a sudden question” and “I don’t care/I am not aware of it” could be found frequently in AD and might be helpful to differentiate between AD and MCI.

In the present study, people with DLB, who indicated fluctuations in attention, impaired executive function, and visual hallucination while the function of episodic memory and orientation were relatively preserved, did not display SARs as frequently as people with AD, in which memory impairment was the core symptom [14, 15]. The underlying mechanism of SARs remains to be elucidated. However, it can be explained by the fact that an impaired theory of mind (ToM) would be seen in the early stages of DLB, compared to AD and MCI [16]. DLB patients sometimes show social behavioral changes at the beginning of the disease [17]. Heitz et al. reported that the people with DLB have impaired ToM, which refers to the capacity to understand someone else’s emotions and mental state [18]. When performing ToM tasks, DLB patients performed worse than people with AD and those with MCI, but better than those with frontotemporal dementia [16]. In our hypothesis, an inverted-U shaped (not linear) relationship between the SARs and frontal lobe dysfunction would exist. Further study aimed at comparing SARs among people with AD, DLB, and frontotemporal dementia will extend the literature on the symptomatology of dementia.

This study was the first study to compare SARs between people with AD and DLB, and revealed that SARs are a typical communication pattern in people with AD. Because it was seen most in the early stage of AD [1], attention to SARs might be helpful for accurate dementia diagnosis. However, this study has several limitations. First, the SARs that we assessed in this study were limited to verbal responses. It is well known that the “attended with” and “head-turning” signs, i.e., turning the head back to glance at the caregiver for help when the patient is taking the neuro-psychological examination, can be seen in people with cognitive impairment [19, 20, 21]. Furthermore, Japanese people sometimes hide their embarrassment behind a smile. Thus, non-verbal behaviors regarding SARs might also need to be examined in future studies. Second, it is possible that SARs may be observed more often in Japanese people because Japan is well known for its social culture of ‘shame,’ and Japanese people are also known to be particularly conscious of how others perceive their actions [22, 23]. Thus, a cultural comparative study on SARs might also produce interesting findings in the future. Third, not only specific functions such as MMSE or FAB scores, but premorbid personalities, insight into their own cognitive decline, or behavioral psychological symptom of dementia are perhaps associated with SARs. These associations should also be examined in future studies.

In this study, we found new evidence regarding early symptoms of dementia. The study’s limitations notwithstanding, our findings contribute to a better understanding of the distress or feelings in people with AD. Medical professionals especially should make an effort to be respectful to people with SARs when they conduct psychological examinations.

## Supporting information

S1 Appendix The Japanese version of the Toritsukuroi Assessment Battery (Ja-TAB). (DOC)

S1 Dataset De-identified data. (XLS)
