# Supplementary material for: Are saving appearance responses typical communication patterns in Alzheimer's disease?
Source: PLoS One. 2018 May 23;13(5):e0197468. doi: 10.1371/journal.pone.0197468 (PMC5965895; doi:10.1371/journal.pone.0197468)
Supplement: S1 Appendix — (DOC) [file pone.0197468.s001.doc]

The Japanese version of the Toritsukuroi Assessment Battery (Ja-TAB)

Date: Patient’s ID: Name of Patient:

Examiner:

Saving appearance responses (SARs) ‘Toritsukuroi’ could be seen when people suspected of having dementia cannot answer correctly under the examination. SARs might delay the early detection and treatment of dementia. When medical staff conducts the ‘Mini-Mental State Examination’ neuropsychological examination, this tool can be used to assess SARs. Please tick the checkbox next to the following verbal responses. Frequency means how many responses were recorded in each domain of the examination. Lastly, please sum up the numbers from all check boxes.

† Do not include erroneous answers or ‘I don’t know.’ by patient.

| Domains of examination | Category | Frequency |
| --- | --- | --- |
| Date Orientation | □ Refutation of sudden question ex. “I can't answer for a sudden question’’ ・・・・・・・  □ Disclosure of traits ex. “Because I don’t like it”, “I’m not good at it” or “I easy get nervous”　・・・・  □ Disclosure of experience  ex. “I usually don’t do such a thing”, “I never do this thing” or “I didn’t do that kind of thing” 　・・・・・・・  □ Demonstration of a slightest hesitation ex. “I could remember until just a while ago” ・  □ Appealing of indifference ex. “I don’t care, I am not conscious of it” ・・・・・・・・・・・・  □ Other ( ) ・・ | □ □ □ □ □  □ □ □ □ □  □ □ □ □ □  □ □ □ □ □  □ □ □ □ □  □ □ □ □ □ |
| Place Orientation | □ Refutation of sudden question ex. “I can't answer for a sudden question’’ ・・・・・・・  □ Disclosure of traits ex. “Because I don’t like it”, “I’m not good at it” or “I easy get nervous”　・・・・  □ Disclosure of experience  ex. “I usually don’t do such a thing”, “I never do this thing” or “I didn’t do that kind of thing” 　・・・・・・・  □ Demonstration of a slightest hesitation ex. “I could remember until just a while ago” ・  □ Appealing of indifference ex. “I don’t care, I am not conscious of it” ・・・・・・・・・・・・  □ Other ( ) ・・ | □ □ □ □ □  □ □ □ □ □  □ □ □ □ □  □ □ □ □ □  □ □ □ □ □  □ □ □ □ □ |
| Registration | □ Refutation of sudden question ex. “I can't answer for a sudden question’’ ・・・・・・・  □ Disclosure of traits ex. “Because I don’t like it”, “I’m not good at it” or “I easy get nervous”　・・・・  □ Disclosure of experience  ex. “I usually don’t do such a thing”, “I never do this thing” or “I didn’t do that kind of thing” 　・・・・・・・  □ Demonstration of a slightest hesitation ex. “I could remember until just a while ago” ・  □ Appealing of indifference ex. “I don’t care, I am not conscious of it” ・・・・・・・・・・・・  □ Other ( ) ・・ | □ □ □ □ □  □ □ □ □ □  □ □ □ □ □  □ □ □ □ □  □ □ □ □ □  □ □ □ □ □ |
| Attention  (Serial-7) | □ Refutation of sudden question ex. “I can't answer for a sudden question’’ ・・・・・・・  □ Disclosure of traits ex. “Because I don’t like it”, “I’m not good at it” or “I easy get nervous”　・・・・  □ Disclosure of experience  ex. “I usually don’t do such a thing”, “I never do this thing” or “I didn’t do that kind of thing” 　・・・・・・・  □ Demonstration of a slightest hesitation ex. “I could remember until just a while ago” ・  □ Appealing of indifference ex. “I don’t care, I am not conscious of it” ・・・・・・・・・・・・  □ Other ( ) ・・ | □ □ □ □ □  □ □ □ □ □  □ □ □ □ □  □ □ □ □ □  □ □ □ □ □  □ □ □ □ □ |
| Repetition | □ Refutation of sudden question ex. “I can't answer for a sudden question’’ ・・・・・・・  □ Disclosure of traits ex. “Because I don’t like it”, “I’m not good at it” or “I easy get nervous”　・・・・  □ Disclosure of experience  ex. “I usually don’t do such a thing”, “I never do this thing” or “I didn’t do that kind of thing” 　・・・・・・・  □ Demonstration of a slightest hesitation ex. “I could remember until just a while ago” ・  □ Appealing of indifference ex. “I don’t care, I am not conscious of it” ・・・・・・・・・・・・  □ Other ( ) ・・ | □ □ □ □ □  □ □ □ □ □  □ □ □ □ □  □ □ □ □ □  □ □ □ □ □  □ □ □ □ □ |
| Verbal Commands  (Tree-stage command) | □ Refutation of sudden question ex. “I can't answer for a sudden question’’ ・・・・・・・  □ Disclosure of traits ex. “Because I don’t like it”, “I’m not good at it” or “I easy get nervous”　・・・・  □ Disclosure of experience  ex. “I usually don’t do such a thing”, “I never do this thing” or “I didn’t do that kind of thing” 　・・・・・・・  □ Demonstration of a slightest hesitation ex. “I could remember until just a while ago” ・  □ Appealing of indifference ex. “I don’t care, I am not conscious of it” ・・・・・・・・・・・・  □ Other ( ) ・・ | □ □ □ □ □  □ □ □ □ □  □ □ □ □ □  □ □ □ □ □  □ □ □ □ □  □ □ □ □ □ |
| Reading and Following | □ Refutation of sudden question ex. “I can't answer for a sudden question’’ ・・・・・・・  □ Disclosure of traits ex. “Because I don’t like it”, “I’m not good at it” or “I easy get nervous”　・・・・  □ Disclosure of experience  ex. “I usually don’t do such a thing”, “I never do this thing” or “I didn’t do that kind of thing” 　・・・・・・・  □ Demonstration of a slightest hesitation ex. “I could remember until just a while ago” ・  □ Appealing of indifference ex. “I don’t care, I am not conscious of it” ・・・・・・・・・・・・  □ Other ( ) ・・ | □ □ □ □ □  □ □ □ □ □  □ □ □ □ □  □ □ □ □ □  □ □ □ □ □  □ □ □ □ □ |
| Recall 3 Objects | □ Refutation of sudden question ex. “I can't answer for a sudden question’’ ・・・・・・・  □ Disclosure of traits ex. “Because I don’t like it”, “I’m not good at it” or “I easy get nervous”　・・・・  □ Disclosure of experience  ex. “I usually don’t do such a thing”, “I never do this thing” or “I didn’t do that kind of thing” 　・・・・・・・  □ Demonstration of a slightest hesitation ex. “I could remember until just a while ago” ・  □ Appealing of indifference ex. “I don’t care, I am not conscious of it” ・・・・・・・・・・・・  □ Other ( ) ・・ | □ □ □ □ □  □ □ □ □ □  □ □ □ □ □  □ □ □ □ □  □ □ □ □ □  □ □ □ □ □ |
| Naming  2 objects | □ Refutation of sudden question ex. “I can't answer for a sudden question’’ ・・・・・・・  □ Disclosure of traits ex. “Because I don’t like it”, “I’m not good at it” or “I easy get nervous”　・・・・  □ Disclosure of experience  ex. “I usually don’t do such a thing”, “I never do this thing” or “I didn’t do that kind of thing” 　・・・・・・・  □ Demonstration of a slightest hesitation ex. “I could remember until just a while ago” ・  □ Appealing of indifference ex. “I don’t care, I am not conscious of it” ・・・・・・・・・・・・  □ Other ( ) ・・ | □ □ □ □ □  □ □ □ □ □  □ □ □ □ □  □ □ □ □ □  □ □ □ □ □  □ □ □ □ □ |
| Writing a Sentence | □ Refutation of sudden question ex. “I can't answer for a sudden question’’ ・・・・・・・  □ Disclosure of traits ex. “Because I don’t like it”, “I’m not good at it” or “I easy get nervous”　・・・・  □ Disclosure of experience  ex. “I usually don’t do such a thing”, “I never do this thing” or “I didn’t do that kind of thing” 　・・・・・・・  □ Demonstration of a slightest hesitation ex. “I could remember until just a while ago” ・  □ Appealing of indifference ex. “I don’t care, I am not conscious of it” ・・・・・・・・・・・・  □ Other ( ) ・・ | □ □ □ □ □  □ □ □ □ □  □ □ □ □ □  □ □ □ □ □  □ □ □ □ □  □ □ □ □ □ |
| Drawing a Pentagon | □ Refutation of sudden question ex. “I can't answer for a sudden question’’ ・・・・・・・  □ Disclosure of traits ex. “Because I don’t like it”, “I’m not good at it” or “I easy get nervous”　・・・・  □ Disclosure of experience  ex. “I usually don’t do such a thing”, “I never do this thing” or “I didn’t do that kind of thing” 　・・・・・・・  □ Demonstration of a slightest hesitation ex. “I could remember until just a while ago” ・  □ Appealing of indifference ex. “I don’t care, I am not conscious of it” ・・・・・・・・・・・・  □ Other ( ) ・・ | □ □ □ □ □  □ □ □ □ □  □ □ □ □ □  □ □ □ □ □  □ □ □ □ □  □ □ □ □ □ |

Total number of the saving appearance responses

( )
